# Ether Vapor in the Practice of Midwifery

**Published:** 1847-07

**Authors:** 


*Application of Ether Vapor to the Practice of Midwifery.*—Professor Simpson has employed ether vapor in the practice of midwifery, and is the first, we believe, who has made the application of this agent. The case was perfectly successful, as the following extract will show:

"A few days ago, Professor Simpson stated to his class that he had practised with entire success the inhalation of sulphuric ether in a case of the most difficult form of labor, and where otherwise the sufferings of the patient would undoubtedly have been extreme. The mother was lame and deformed. At a former accouchement, the labor lasted three or four days, and from the necessarily protracted use of instruments, the patient's agonies were very great. On the present occasion, Dr. Simpson had previously determined to avoid, if possible, the use of all instruments, and to attempt to extract the infant by the feet. He expected to be aided in this by the use of the ether inhalation. Accordingly, when labor had set in for a few hours, the patient was put under the influence of ether, and in a few minutes the child was turned and extracted, while the mother was altogether unconscious of the operation, and that, too, although the delivery was rendered excessively difficult, by the degree of compression to which the child's head required to be subjected. On afterwards awakening, or passing from her 'etherealized' condition to the state of common consciousness, one of the first circumstances of which the patient became aware, was the noise attendant on preparing a bath to resuscitate the infant. A remarkable circumstance pointed out in the case by Dr. Simpson was, that whilst breathing the ether, the labor pains or throes continued, and yet the mother (to speak paradoxically) felt no pains. We hear she is rapidly recovering. This is, we believe, the first instance in which this new and extraordinary agent has been employed in the practice of midwifery.—*London Medical Gazette.*

